# GroScore: Automated Scoring of Protein–Protein Interactions Using Bidirectional Steered Molecular Dynamics

**DOI:** 10.1021/acs.jcim.6c01771

**Published:** 2026-10-02

**Authors:** Jan Walther Perthold, Dražen Petrov, Chris Oostenbrink

**Affiliations:** † Independent Researcher, Vienna 1130, Austria; ‡ Institute for Molecular Modeling and Simulation, Department of Natural Sciences and Sustainable Resources, 27270 BOKU University, Muthgasse 18, Vienna 1190, Austria; § Christian Doppler Laboratory for Molecular Informatics in the Biosciences, BOKU University, Muthgasse 18, Vienna 1190, Austria

## Abstract

GroScore is an open-source Python toolkit that automates the scoring of protein–protein structures using bidirectional steered molecular dynamics (SMD) simulations with explicit solvent. Building on a previously published unidirectional pulling approach, the current version introduces bidirectional pulling (unbinding and rebinding), interface cutout for reduced computational cost, support for three force fields (AMBER19SB, CHARMM36m, GROMOS 54A8), and automated handling of small molecules and noncanonical amino acids (using OpenFF), structural ions, and crystal waters. GroScore integrates with GROMACS and SLURM for high-performance computing and is validated on protein–protein affinity benchmarks, showing significant correlation between computed scores and experimental binding affinities across all tested force fields. The software is freely available at https://github.com/jwperthold/GroScore under the MIT License.

## Graphical abstract

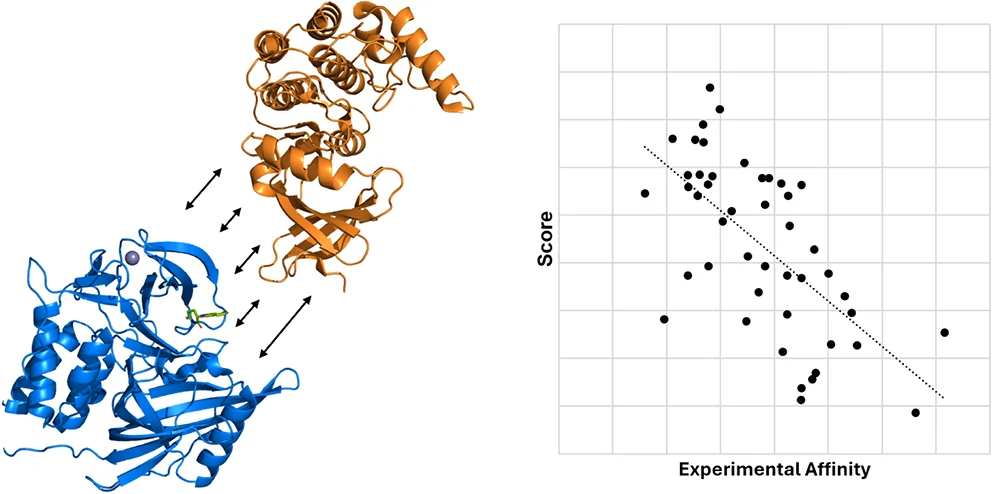

## Introduction

Protein–protein interactions (PPIs) are central to virtually all cellular processes, making their structural characterization essential for understanding biology and developing therapeutics. Computational docking methods and co-folding approaches can generate large numbers of candidate complex structures, but reliably quantifying the strength of binding as well as distinguishing near-native poses from non-native structures remains a major challenge.

Recent years have seen a deep-learning-driven revolution in the modeling of protein assemblies. AlphaFold-Multimer,^1^ its diffusion-based successor AlphaFold 3,^2^ the all-atom variant of RoseTTAFold,^3^ and a growing ecosystem of openly available successors such as Boltz^4,5^ now produce plausible 3D models of protein–protein complexes directly from sequence, often within minutes. These predictors also act as *de facto* scoring functions through their internal confidence metrics, most prominently the interface predicted template modeling score (ipTM) and the closely related pDockQ score, which combines interface pLDDT (predicted local distance difference test) with the size of the predicted contact patch. Both metrics correlate broadly with model quality and are now widely used to rank candidate complexes, including in proteome-scale interaction screens.^6^ They are, however, not sufficient as standalone scoring solutions: documented failure modes include false positives for non-interacting pairs and a marked drop in reliability for antibody−antigen complexes, transient interfaces, and other systems with limited co-evolutionary signal. Recent work questions whether AI co-folding methods generalize beyond memorization of training data,^7^ and quantitative analyses show that reported protein−ligand benchmark correlations can be substantially inflated by data leakage.^8^ In narrower, data-rich domains such as the prediction of the effect of antibody mutations on antigen binding (ΔΔG), supervised models can reach high apparent accuracy (Pearson correlations up to 0.87 on AB-Bind), but this performance has been shown to stem largely from overtraining on limited experimental data. Correlations have been shown to drop by ∼63% once train–test sequence-identity cutoffs are imposed, and orders of magnitude more experimental data are estimated to be needed for generalizable prediction.^9^ Even when sampling is no longer the bottleneck, reliable scoring of candidate poses therefore remains a persistent open problem, as systematically demonstrated in successive CAPRI scoring experiments and in the most recent CASP15-CAPRI joint assessment.^10,11^ Dedicated post hoc scoring functions, developed and benchmarked against this problem for decades, span a broad methodological spectrum: empirical, force-field-based combinations of energy terms such as those underlying HADDOCK^12^ and pyDock;^13^ knowledge-based statistical potentials such as SOAP-PP;^14^ integrative approaches that combine energetic terms with co-evolutionary and topological information, such as iScore;^15^ deep-learning approaches that learn interface patterns directly from structural data, exemplified by the DeepRank family of methods;^16^ end-point free-energy methods such as MM/PBSA that re-rank docking poses using a continuum-solvent approximation;^17^ and path-sampling MD-based scoring, pioneered in the protein–protein context by Hou et al. with coarse-grained equilibrium umbrella sampling,^18^ extended to an atomistic level by Kingsley et al. with steered MD and potential-of-mean-force calculations,^19^ and systematically developed by the Zacharias group through umbrella sampling along a dissociation coordinate^20^ and through repulsive-scaling replica-exchange simulations that couple refinement with free-energy-based scoring.^21–23^ A related strategy was reported by Wang et al., who dissociate complexes by ensemble metadynamics along an intermolecular distance coordinate and average the resulting free-energy surfaces to obtain a dissociation free energy, validated across protein–protein and protein–ligand complexes.^24^ The reader is also referred to recent reviews for comprehensive comparisons.^25,26^ Each class entails characteristic trade-offs. AI-derived confidence scores and machine-learning scorers are fast but inherit the biases and redundancies of their training sets and generalize poorly to antibody−antigen complexes, transient interfaces, and other systems with limited co-evolutionary signal underrepresented in the PDB. Empirical and statistical functions are parameterized against narrow benchmarks and remain sensitive to the precise geometry of the input pose. End-point free-energy methods sample only narrow conformational regions around that input and rely on continuum approximations of the interfacial water network. Explicit-solvent MD, by contrast, is force-field-based and in principle transferable across any system class and explicitly samples the unbinding (or rebinding) process rather than relying on its end points. It is therefore a natural, in principle generally applicable, complement to rapid AI-based pose generation or scoring in a hybrid workflow in which generative models propose candidate structures and physics-based simulations critically reevaluate them.

We previously introduced GroScore as a nonequilibrium, explicit-solvent steered MD (SMD) approach to protein–protein pose scoring, in which the binding partners are pulled apart along an unbinding coordinate and the resulting work distributions are converted into a binding score via Jarzynski-type exponential averaging.^27–29^ Three limitations restricted its broader use: (i) the unidirectional pulling protocol suffers from the well-documented statistical bias of exponential work averaging, in which the estimator is dominated by rare low-work trajectories;^30,31^ (ii) computed scores correlated strongly with the buried surface area of the input complex, partially conflating geometric size with binding strength; and (iii) the computational cost of fully solvated, atomistic SMD on entire protein–protein complexes combined with a workflow that required substantial manual setup limited the method to a small number of structures and discouraged routine adoption.

Here, we present GroScore, a production-ready, open-source implementation of an improved protein–protein scoring pipeline that orchestrates simulations via the efficient, widely adopted, and fully open-source GROMACS software.^32^ Building on the original method,^27^ the current version incorporates two key improvements: (1) bidirectional pulling, in which both protein unbinding and rebinding are simulated to overcome exponential averaging bias and reduce correlation with buried surface area; and (2) interface cutout, in which only atoms proximal to the protein–protein interface are retained for simulation, dramatically reducing computational cost. A similar approach of only simulating the most important region of a system has been successfully used for relative free energy calculations of small molecules and protein mutations.^33–36^ In addition, the pipeline now supports three force fields, automated handling of small molecules, noncanonical amino acids, and structural ions. The software is validated on protein–protein affinity benchmarks with known experimental binding affinities.

## Implementation

### Simulation Pipeline

GroScore automates the complete workflow from an input PDB file (either an experimentally determined or a computationally predicted structure) to final binding affinity scores (Figure 1). The pipeline comprises: (i) structure preparation and GROMACS^32^ topology generation; (ii) optional interface cutout extracting only residues within 2.0 nm of the protein–protein interface; (iii) solvation with 0.15 M NaCl in a dodecahedron box with a minimum protein-wall distance of 1.5 nm and energy minimization; (iv) an initial equilibration to generate distance restraints; and (v) *N* independent pull/push cycles (default: 5), each starting from a fresh equilibration with new random velocities to ensure statistical independence. Because MD is chaotic, independently seeded cycles decorrelate rapidly, so an ensemble of short cycles samples the binding/unbinding process effectively.^37^

**Figure 1:**
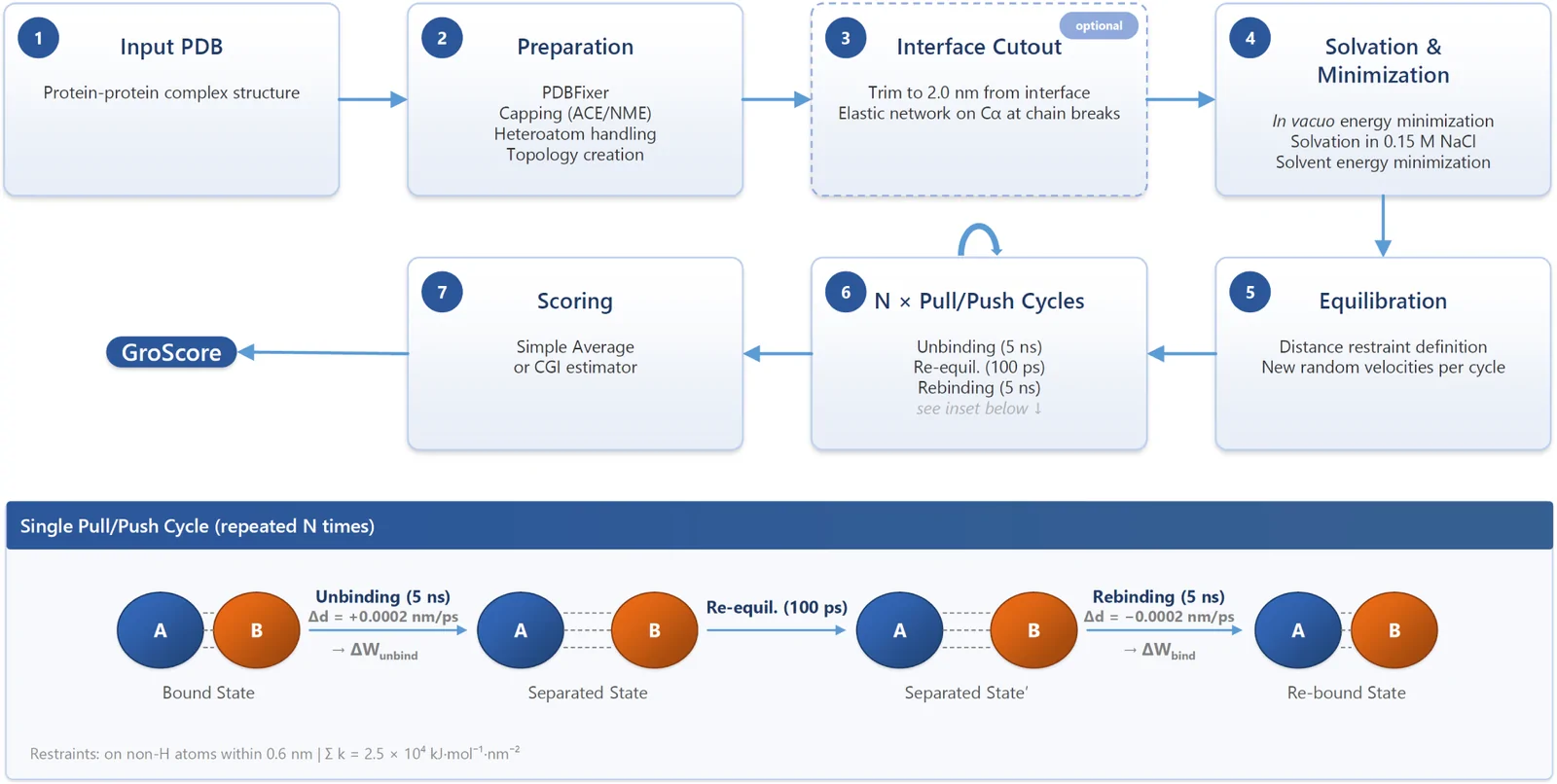
Overview of the GroScore simulation pipeline. Starting from an input PDB file, structures undergo automated preparation (fixing, capping, optional interface cutout), solvation and energy minimization, followed by *N* independent pull/push cycles each initiated from fresh equilibration with new random velocities. Final scores are computed using two statistical methods.

### Interface Cutout

To reduce simulation box sizes and computational cost, GroScore can extract only residues within a cutoff distance (default: 2.0 nm) of the protein–protein interface. Elastic network distance restraints (0.4–0.9 nm range, 250 kJ·mol^–1^·nm^–2^) are applied to Cα atoms at artificial chain breaks introduced by this procedure, maintaining structural integrity.

### Force Field Support

GroScore supports different force fields: AMBER19SB with OPC3 water (default, 3-point), AMBER19SB with OPC water (4-point),^38–40^ CHARMM36m with TIP3P,^41–43^ and GROMOS 54A8 with SPC.^44–46^ All use PME electrostatics,^47^ all-bonds constraints,^48,49^ and hydrogen mass repartitioning for stable 4 fs time steps.^50,51^ Terminal capping is force-field-specific: ACE/NME for AMBER19SB, ACE/COOH for CHARMM36m and GROMOS 54A8.

### Heteroatom Handling

Structural metal ions are automatically detected and coordination is maintained via topology-level harmonic restraints. For AMBER19SB, small molecules can be parameterized using the Open Force Field^52,53^ (Sage 2.2.1) with Open Babel^54^ bond perception and RCSB template fallback.^55^ Noncanonical amino acids (e.g., phosphorylated or methylated residues) are handled via a hybrid parameterization: a capped tripeptide fragment is built from the PDB coordinates, OpenFF Sage assigns charges and bonded parameters for the modified side chain, while backbone atoms retain force field parameters from the parent residue. For GROMOS 54A8, post-translationally modified amino acids are natively supported using the Vienna-PTM parameter set.^81,82^ Crystal waters from PDB structures are preserved at their crystallographic positions.

### Bidirectional Pulling

Distance restraints are defined between all non-hydrogen atom pairs (one from each binding partner) whose interatomic distance in the initial complex structure is ≤0.6 nm. The sum of equally partitioned force constants is 2.5 × 10^4^ kJ·mol^–1^·nm^–2^. Unbinding is simulated for 5 ns by increasing restrained distances at 0.0002 nm·ps^–1^ (up to 1.0 nm displacement). At this separation, work profiles plateau, as demonstrated earlier.^27^ After a 100 ps re-equilibration of the restrained unbound state, binding is simulated in reverse for 5 ns. Note that the protein structures remain restricted in their motion due to the protein–protein restraints and the elastic network, and it is the fast water−protein interactions that readily equilibrate during this short re-equilibration. This bidirectional approach overcomes the numerical bias of exponential work averaging and substantially reduces the anticorrelation between scores and buried surface area observed with unidirectional pulling. The restraining of all non-hydrogen atom pairs in the interface area restricts relative rotation of the proteins and allows for correct rebinding during the short simulation time (see below and Figure S9 in the Supporting Information (SI)). We note that this separation strategy is best suited to complexes with well-defined, separable interfaces. For intertwined interfaces in which the binding partners wrap around one another, larger separations and different reaction coordinates could be required, analogous to center-of-mass pulling.

### Scoring Methods

Two scoring methods are provided: (1) the simple average of pull and push works across all cycles,^30^ which is unbiased when the forward and reverse work distributions are Gaussian with equal variance; and (2) the Crooks Gaussian Intersection (CGI) estimator,^31^ which fits independent Gaussians to the unbinding and rebinding work distributions and extracts Δ*G* from their intersection. CGI is more statistically efficient when the distributions have unequal variance and is the recommended choice when ≥20 independent cycles are available. Bootstrap confidence intervals (CI) are computed for both methods (*N* = 50,000 resamples). Score files are updated after each completed cycle, enabling convergence monitoring.

## Validation

The new implementation of GroScore was initially validated on the highly curated HADDOCK protein–protein affinity benchmark,^56,57^ comprising 46 protein–protein complexes with experimentally determined binding affinities spanning a range of approximately 8 p*K*_d_ units. Simulations were performed with all three supported force fields with and without the interface cutout mode, using simple averaging of the unbinding and binding work estimates. Benchmark results with 5 independent pull/push cycles are shown in Figure 2. Significant positive correlations (Pearson *r* = 0.51–0.66) of calculated scores to experimental binding affinities were obtained for all tested force fields (see Figures S1 to S4 in the SI). Moreover, all force fields showed similar scoring performance and the correlation results of cutout mode vs. no-cutout were virtually identical. Furthermore, the per-complex scores obtained in cutout and no-cutout modes are themselves strongly correlated (Pearson *r* = 0.93, 0.96, 0.93, and 0.87 for AMBER19SB/OPC, AMBER19SB/OPC3, CHARMM36m/TIP3P, and GROMOS 54A8/SPC, respectively), confirming that the cutout procedure preserves the scores of individual complexes, not merely the aggregate correlation. AMBER19SB/OPC3 with cutout mode was chosen as the standard setting for the workflow and for all following simulations because of performance and cross-compatibility with OpenFF. Backbone RMSDs of the rebound state relative to the bound starting structure are comparable between cutout and no-cutout simulations across all four force-field/water-model combinations, with ≥95% of trajectories remaining within 5 Å of the starting structure (Figure S9), confirming that the cutout procedure does not compromise structural integrity. Cutout mode was therefore determined to be a viable strategy to save computational time. Small differences in the simulation wall-times without cutout mode between the force fields could be explained by the different real-space cutoffs used by each force field (1.0 nm for AMBER19SB, 1.2 nm for CHARMM36m, 1.4 nm for GROMOS 54A8 following each force field’s parameterization convention), which scale the neighbor-list size and short-range nonbonded cost. A second, competing factor is the number of interacting particles, which is lower for the united-atom GROMOS 54A8 force field than for the all-atom AMBER19SB and CHARMM36m. This reduced particle count can explain why GROMOS 54A8 remains competitive and in cutout mode faster than CHARMM36m, despite its larger cutoff. Figures S5–S8 in the SI show the convergence of the scoring performance as a function of the number of cycles performed. Although Figures S5–S8 show that 5 cycles already give converged correlations for the HADDOCK benchmark, more cycles in combination with the CGI estimator can resolve small effects that the simple average cannot (see molecular-glue results below).

**Figure 2:**
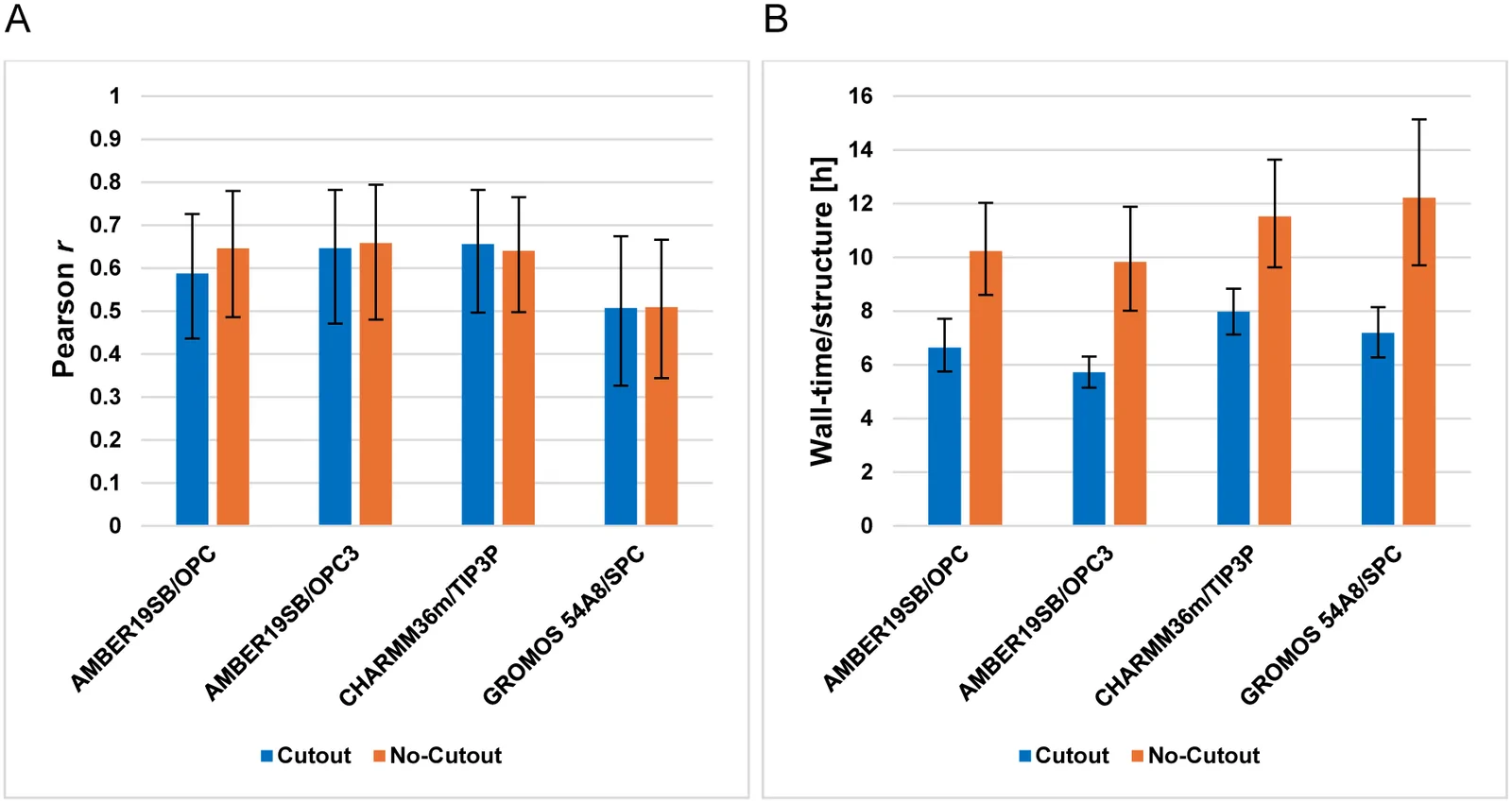
(A) Correlation of computed scores to experimental binding affinities for the HADDOCK protein–protein affinity benchmark. (B) Average simulation wall-time per structure for the benchmark with a single NVIDIA A40 GPU (5 independent cycles). Error bars indicate 95% CI.

Next, we tested the implementation with the much larger aggregate PPB-Affinity benchmark dataset.^58^ We performed scoring calculations using cutout mode only for wild-type complexes with AMBER19SB + OpenFF Sage/OPC3 and 5 independent cycles. Out of 2529 complexes, 2431 were successfully simulated (96% success rate). The largest reason for failure was broken loops detected at the interface area (53 complexes, more than half of the failed simulations). For the overall dataset, we obtained a weak but significant correlation (Pearson *r* = 0.16, Spearman ρ = 0.24). We observed large differences in scoring performance for the different source datasets (see Table 1).

**Table 1:** Scoring Performance (AMBER19SB + OpenFF Sage/OPC3, 5 Cycles) for the Different PPB-Affinity Source Datasets^a^

Source	N	Pearson *r*	Spearman ρ	σ(p*K*_d_)	mean(p*K*_d_)
Affinity Benchmark v5.5	86	0.40 [0.25, 0.57]	0.44 [0.25, 0.62]	2.15	7.76
SKEMPI v2.0	296	0.20 [0.07, 0.36]	0.34 [0.23, 0.44]	2.18	7.66
PDBbind v2020	1545	0.20 [0.15, 0.26]	0.28 [0.23, 0.32]	1.79	6.89
SAbDab	495	0.17 [0.09, 0.25]	0.17 [0.08, 0.25]	1.43	8.27
ATLAS	9	–0.36 [−0.85, 0.21]	–0.27 [−0.98, 0.48]	0.86	4.69
All combined	2431	0.16 [0.12, 0.20]	0.24 [0.20, 0.27]	1.88	7.29

a Values in brackets indicate 95% CI. σ­(p*K*_d_) indicates the standard deviation in experimental p*K*_d_ in the dataset.

The performance gradient across the five source datasets is informative. The strongest correlation is observed for the Affinity Benchmark v5.5 (Pearson *r* = 0.40), which is the descendant of the same HADDOCK curated benchmark used in the initial validation above (the 46-complex set is a subset of these 86 cases) and remains the gold standard for non-redundant, high-resolution complexes with carefully cross-validated binding affinities measured by direct biophysical methods.^59,60^ Performance drops by roughly half for the larger but less stringently curated PPI subset of PDBbind v2020 (*r* = 0.20),^61,62^ and for SKEMPI v2.0 wild-type structures (*r* = 0.20),^63^ which were originally curated to support ΔΔ*G* mutation studies rather than absolute affinity prediction and are dominated by a handful of well-studied complex families (proteases/inhibitors, antibody−antigen). The SAbDab antibody−antigen subset^64^ gives only marginal correlation (*r* = 0.17) despite its size; this reflects two confounding factors. First, antibody−antigen complexes pose specific structural challenges: CDR loops, especially the highly variable H3 loop, are frequently incomplete or partially disordered in deposited crystal structures, and their bound conformation often differs substantially from the unbound state. Even where the loops are resolved at the interface (and so pass our broken-loop filter), unmodeled or low-occupancy regions away from the interface may still affect equilibration and scoring. Second, antibody−antigen affinities span a comparatively narrow dynamic range relative to the broader PPI universe, compressing the signal available to any scoring function. The same dynamic-range issue, in extreme form, dominates the ATLAS TCR−pMHC subset,^65^ where only nine wild-type complexes met our processing criteria and where the experimentally measured affinities span barely two p*K*_d_ units. The negative point estimate of correlation here should be read as essentially uninformative (a 95% CI that crosses zero in both Pearson and Spearman) rather than as a real signal of anti-correlated scoring. Figure S10 in the SI shows the GroScore-predicted vs. experimental p*K*_d_ values for each source dataset, with subset-specific linear fits, visualizing the trends summarized in Table 1.

To illustrate the improved ability of GroScore to discriminate near-native poses from incorrect poses, we scored targets of the CAPRI Score_set^66^ using the updated workflow with a single simulation cycle. A total of 49 (including 13 high-quality and 23 medium-quality) near-native poses were ranked within the Top-10 of each target, compared to 40 (including 9 high-quality and 21 medium-quality) with the original unidirectional setup with the same amount of simulation time.^27^ Detailed results are given in Figure S11 (scatterplots I-RMSD vs. score) and Table S1 (CAPRI evaluation) in the SI. The native, experimentally determined structures of the investigated targets and poses of set T32 were scored using an NVIDIA RTX 5080 GPU, resulting in a (single-cycle) performance of 56 poses/day and 53 poses/day, respectively, showcasing the scoring efficiency of GroScore on consumer-grade hardware.

Last, we tested the small-molecule implementation in the context of proximity-inducing compounds. A set of 19 ternary complexes, 14 molecular glues (MGs) and 5 proteolysis-targeting chimeras (PROTACs), was scored in the presence and absence of the inducer. The small-molecule inducer is assigned to one of the two binding partners according to its chain identifier and is otherwise treated identically to a protein residue. Interface distance restraints are generated between the inducer atoms and atoms of the opposing partner within the 0.6 nm cutoff, so that the inducer participates in the pulling coordinate exactly as interface protein atoms do, while no restraints tether it to its host partner; its association with the host is therefore maintained only by the physical force field. The reaction coordinate consequently separates the partner from the inducer-bound complex. This assignment is well-defined for glues that occupy a pocket on one partner, but is inherently ambiguous for linker-bridging PROTACs, consistent with the larger variance observed for those systems. The GroScore differences (glued – non-glued) from both the simple average over 5 cycles and the CGI estimator over 30 cycles are shown in Figure 3. With CGI, a significant negative shift indicating ternary-complex stabilization by the small molecule was obtained for 13 of the 19 systems. The six remaining cases fall into three categories that are consistent with the experimentally known affinity profiles of these systems.

**Figure 3:**
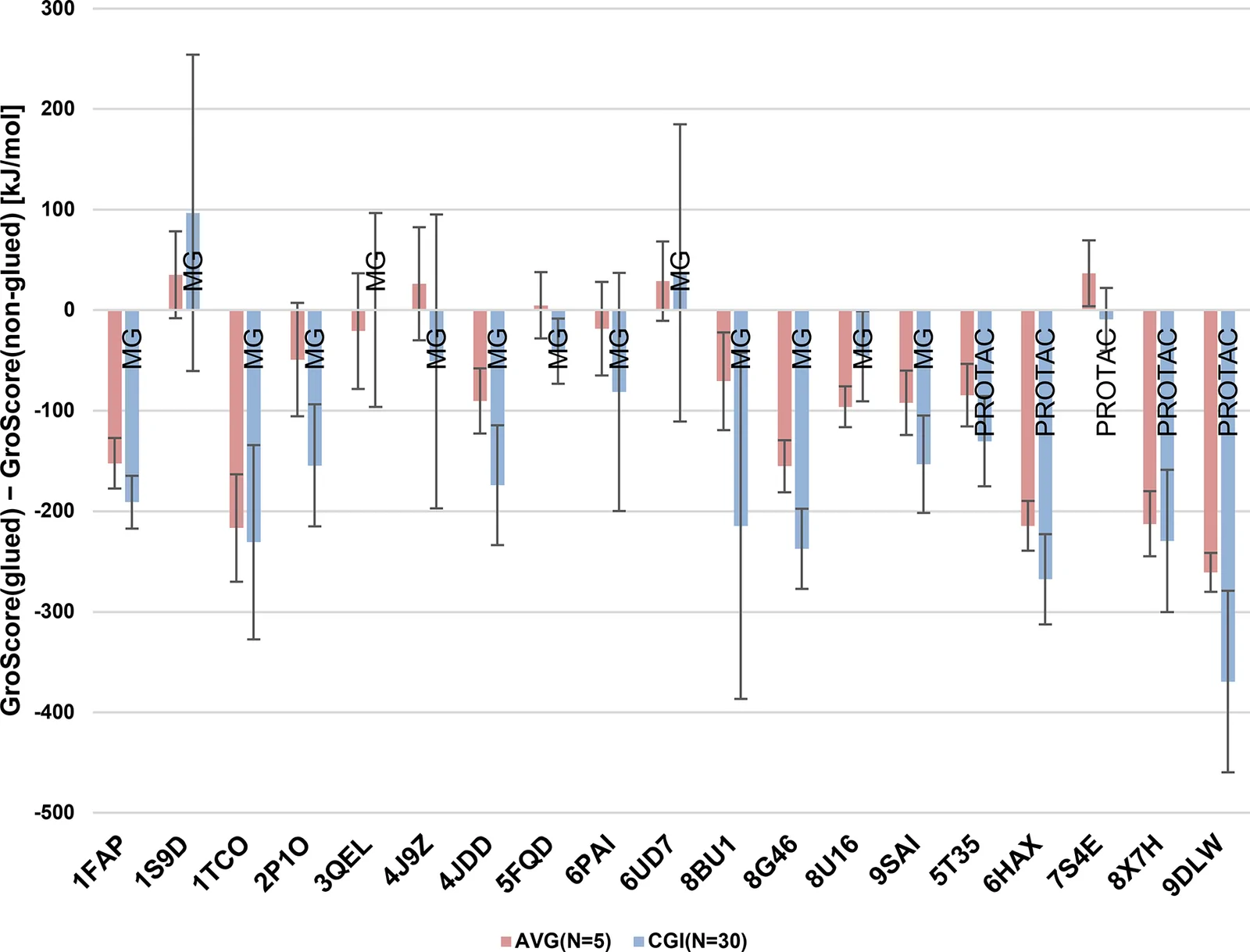
GroScore differences (glued – non-glued) for 19 small-molecule-induced PPIs (14 MGs and 5 PROTACs), computed using the simple average of pull and push works over 5 independent cycles (red, AVG, *N* = 5) and the Crooks Gaussian Intersection estimator over 30 independent cycles (blue, CGI, *N* = 30). Error bars indicate 95% bootstrap confidence intervals.

The first category comprises complexes with substantial protein–protein affinity already in the absence of the inducer. The NMDA receptor amino-terminal-domain heterodimer GluN1/GluN2B (PDB 3QEL) has an intrinsic K_d_ of 0.7–1 μM that ifenprodil only modestly enhances; calmodulin binds the SK channel CaM-binding domain (PDB 4J9Z) in the nanomolar range with the positive modulator NS309 contributing a comparatively small additional stabilization; and both RBM39/DCAF15 sulfonamide-glue complexes (PDB 6UD7, 6PAI) show a detectable basal affinity with a glue-induced enhancement of less than two orders of magnitude.^67^ In all of these cases the difference between binary and ternary affinity is at or near the resolution limit of GroScore.

The second category is illustrated by the brefeldin-A-mediated ARF1/Sec7 complex (PDB 1S9D), in which the crystallographic geometry corresponds to an abortive intermediate of the GDP/GTP exchange reaction.^68,69^ There is no stable ARF1/Sec7 complex without brefeldin A, but the input structure is already shape-complementary and the simulation samples this preorganized interface as a stable contact, producing a slightly positive, yet nonsignificant GroScore difference. This points to a more general limitation: GroScore reports on the free energy of dissociating the input geometry, not on the free energy of forming the complex from the unbound monomers.

The third nonsignificant case is the SMARCA2/VHL PROTAC ACBI1 (PDB 7S4E), reported as one of the most cooperative SMARCA2 degraders. PROTACs differ mechanistically from glues in that the inducer mediates association through two distinct warhead pockets rather than through a localized protein–protein interface; the protein–protein contact alone is intrinsically weak. The four other PROTACs in the set (PDB 5T35, 6HAX, 8X7H, 9DLW) nonetheless gave significant negative GroScore differences.

Two further cases illustrate the practical benefit of the CGI estimator over the simple average. For both the TIR1/auxin/IAA7 system (PDB 2P1O) and the CRBN/lenalidomide/CK1α complex (PDB 5FQD) the simple average over 5 cycles gave a nonsignificant difference, whereas the CGI estimator over 30 cycles resolved a clearly significant glue effect. This improvement combines two effects: additional cycles reduce the variance of the estimate, and the CGI estimator additionally exploits the asymmetry between forward (unbinding) and reverse (rebinding) work distributions through their Gaussian intersection point.^31^ The CGI estimator is therefore our recommended choice whenever ≥20 independent cycles are computed. We note that CGI CIs are in some cases wider than those of the simple average; this reflects that CGI depends on the intersection of fitted forward and reverse work distributions, and thus on their variances, which converge more slowly than the mean and dominate the residual uncertainty even at 30 cycles.

## Conclusion

We have presented GroScore as a production-ready open-source toolkit for protein–protein scoring, automating the complete workflow from input structure to binding score using bidirectional steered molecular dynamics with explicit solvent. Building on the original unidirectional method, the implementation now incorporates bidirectional pull/push cycles to mitigate the bias of exponential work averaging, an interface-cutout mode that substantially reduces computational cost without measurable loss of accuracy, support for three established protein force fields, and automated parameterization of small molecules, noncanonical amino acids, structural ions, and crystal waters. It is available as a fully open source software package, to support use over a broad range of applications. Validation on the curated HADDOCK affinity benchmark and the larger and more heterogeneous PPB-Affinity aggregate shows significant correlations with experimental binding data across diverse system classes. Additionally, GroScore was tested on the CAPRI Score_set, where bidirectional pulling recovered 49 near-native poses in the Top-10 versus 40 for the original unidirectional method.

Two caveats are worth emphasizing. First, GroScore is by design a scoring scheme rather than a rigorous binding free-energy method: sampling is restricted to the direct interaction contributions, and the restraint corrections required for absolute standard-state Δ*G* estimation are deliberately omitted, trading quantitative affinities for tractability and broad applicability. Second, the correlations across the PPB-Affinity subsets, while statistically significant, are weak in absolute terms (Pearson *r* ≈ 0.2–0.4), reflecting the approximations inherent in the scoring scheme, force-field limitations, the finite time scale of nonequilibrium sampling, and the inherent heterogeneity of literature-derived affinity data. These observations underscore that structure-based affinity prediction remains a difficult problem and motivate continuing work on improved sampling schemes and the treatment of binding-induced conformational change.

## Supplementary Material



## Data Availability

GroScore is freely available under the MIT License at https://github.com/jwperthold/GroScore and is archived at Zenodo (release v1.4, DOI: 10.5281/zenodo.22686987 ). The repository includes all simulation settings, benchmark setup scripts, and documentation. GroScore requires Python 3.10,^70^ GROMACS 2026,^32^ and a SLURM-based^71^ HPC environment or a local GPU machine. A conda environment specification is provided for all Python dependencies (NumPy,^72^ SciPy,^73,74^ OpenMM,^75,76^ PDBFixer,^77^ RDKit,^78^ and OpenFF Toolkit^79,80^ ).

## Notes

Scatterplots of predicted vs. experimental p*K*_d_ values after linear fit for the HADDOCK Protein–Protein Affinity Benchmark for AMBER19SB/OPC, AMBER19SB/OPC3, CHARMM36m/TIP3P, and GROMOS 54A8/SPC (Figures S1–S4); convergence of GroScore prediction performance as a function of the number of independent pull/push cycles for the same four force-field/water-model combinations on the HADDOCK benchmark (Figures S5–S8); protein backbone RMSD distributions of the rebound state with respect to the bound state for cutout vs. no-cutout simulations (Figure S9); scatterplot of GroScore-predicted vs. experimental p*K*_d_ for the PPB-Affinity source datasets (Figure S10); scatterplots of GroScore vs. I-RMSD for the CAPRI Score_set targets (Figure S11); and CAPRI evaluation of the Score_set benchmark (Table S1) (PDF)

## Abbreviations

AI: artificial intelligence
CAPRI: Critical Assessment of PRedicted Interactions
CASP: Critical Assessment of protein Structure prediction
CDR: complementarity-determining region
CGI: Crooks Gaussian Intersection
CI: confidence interval
HPC: high-performance computing
ipTM: interface predicted template modeling score
I-RMSD: interface root-mean-square deviation
MD: molecular dynamics
MG: molecular glue
MM/PBSA: molecular mechanics Poisson–Boltzmann surface area
pDockQ: predicted DockQ
pLDDT: predicted local distance difference test
PME: particle mesh Ewald
PPB: protein–protein binding
PPI: protein–protein interaction
PROTAC: proteolysis-targeting chimera
RMSD: root-mean-square deviation
SMD: steered molecular dynamics
TCR–pMHC: T-cell receptor/peptide–major histocompatibility complex
