# Supplementary material for: GroScore: Automated Scoring of Protein–Protein Interactions Using Bidirectional Steered Molecular Dynamics
Source: J Chem Inf Model. 2026 Oct 2;66(20):13032–40. doi: 10.1021/acs.jcim.6c01771 (PMC13715378; doi:10.1021/acs.jcim.6c01771)
Supplement: Supplementary file 1 [file ci6c01771_si_001.pdf]

Supporting Information

GroScore: Automated Scoring of Protein–Protein Interactions Using Bidirectional Steered Molecular Dynamics

Jan Walther Perthold[§,*], *Dražen Petrov*[†], *Chris Oostenbrink*[†,‡]

[§] Independent Researcher, 1130 Vienna, Austria

[†] Institute for Molecular Modeling and Simulation, Department of Natural Sciences and Sustainable Resources, BOKU University, Muthgasse 18, 1190 Vienna, Austria

‡ Christian Doppler Laboratory for Molecular Informatics in the Biosciences, BOKU University, Muthgasse 18, 1190 Vienna, Austria

* jan@ackergarten.at

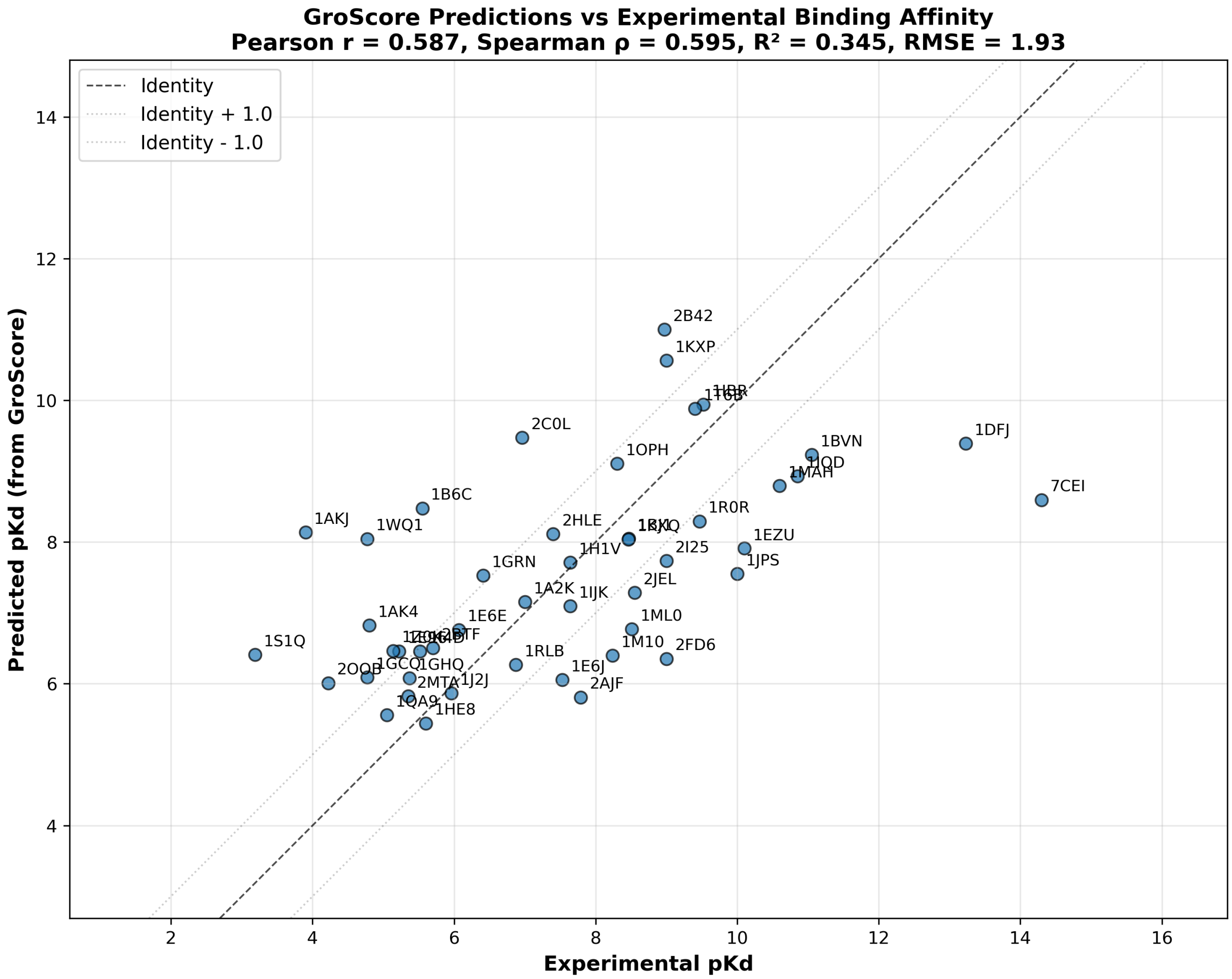

Figure S1. Scatter plots of predicted pK_d values computed from GroScores (cutout-mode, 5 independent cycles) after a linear fit vs. experimental pK_d values for the HADDOCK Protein–Protein Affinity Benchmark (46 structures) for AMBER19SB/OPC (pK_d = -0.0155 × GroScore + 4.2704).

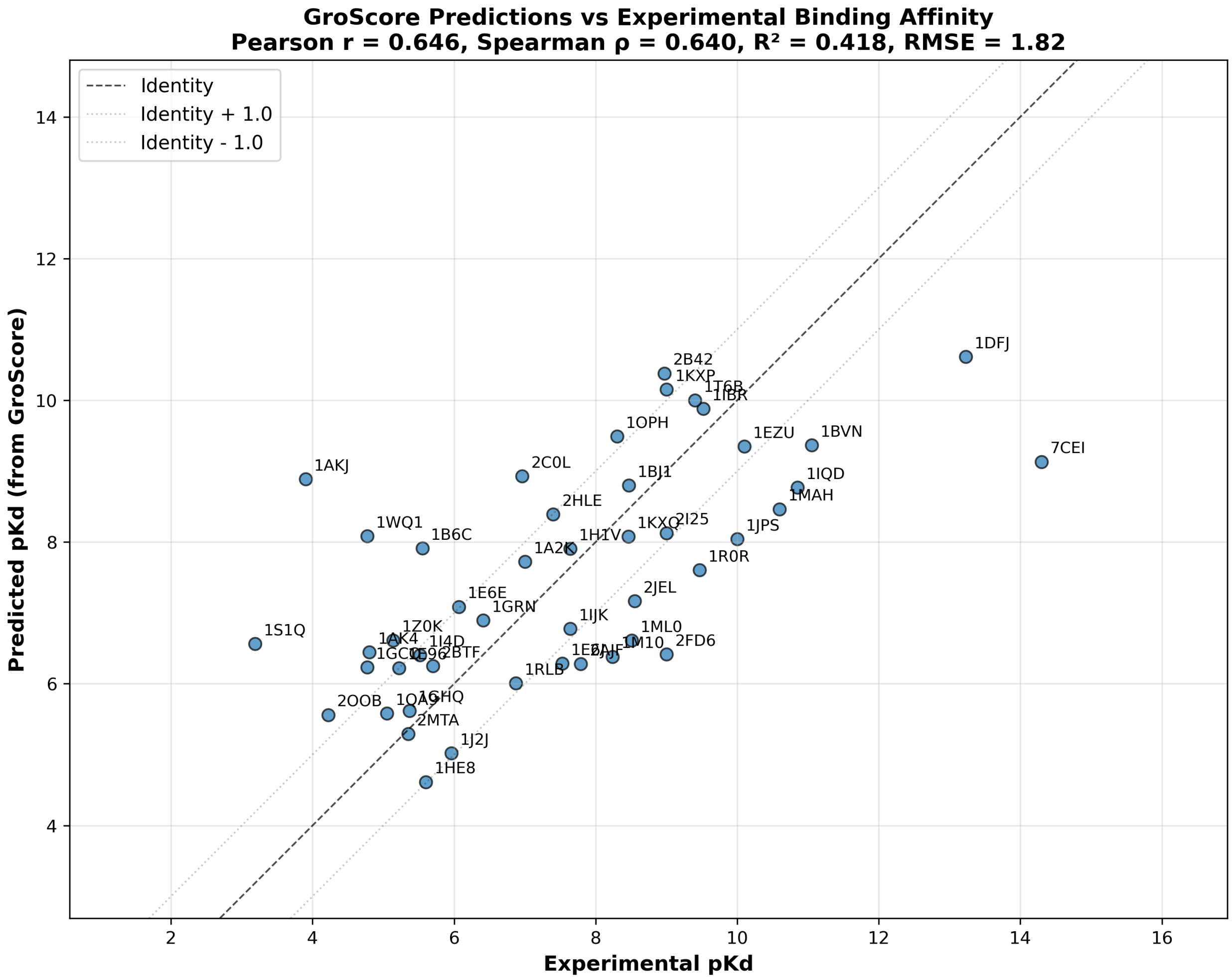

Figure S2. Scatter plots of predicted pK_d values computed from GroScores (cutout-mode, 5 independent cycles) after a linear fit vs. experimental pK_d values for the HADDOCK Protein–Protein Affinity Benchmark (46 structures) for AMBER19SB/OPC3 ($pK_d = -0.0176 \times \text{GroScore} + 3.4513$).

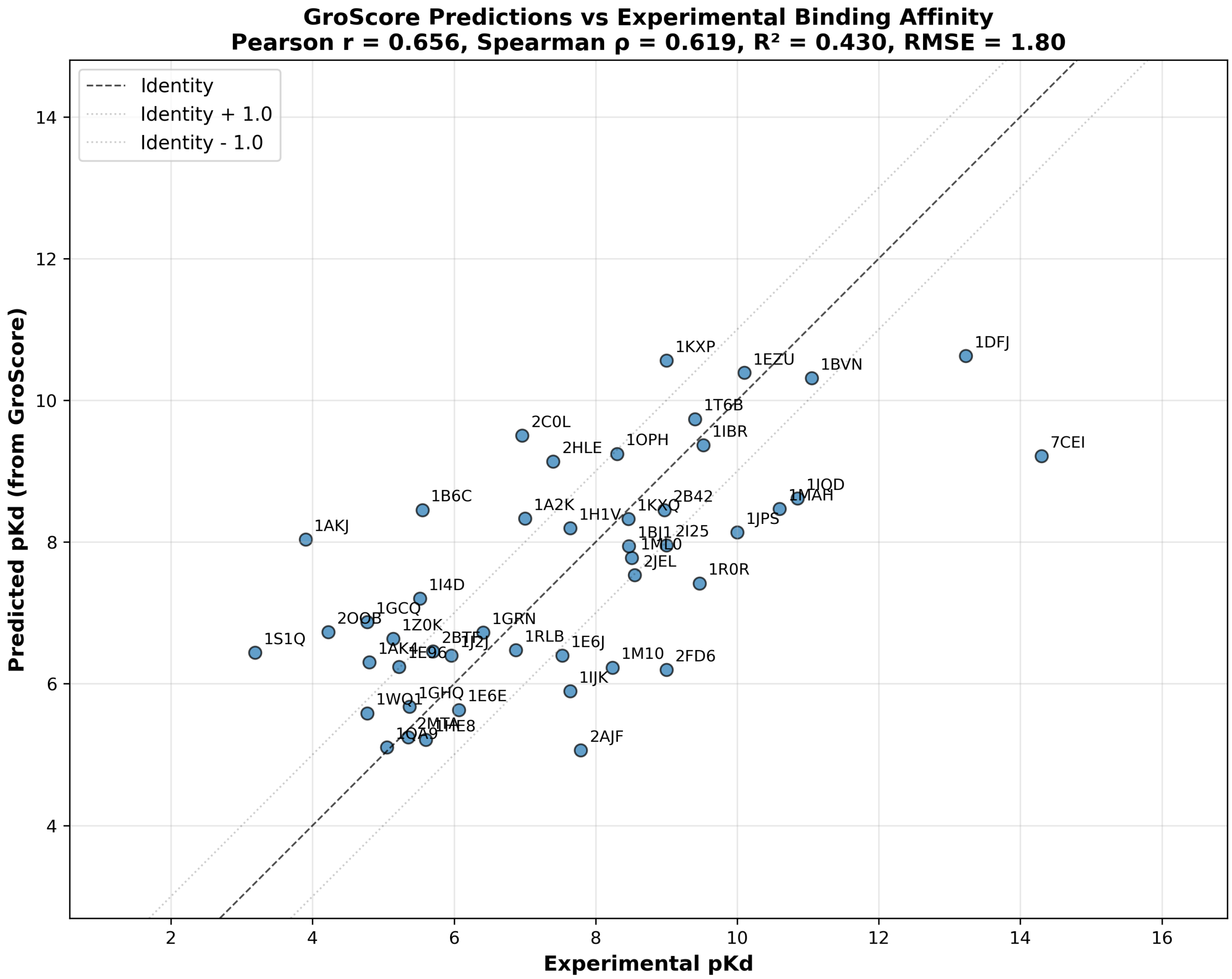

Figure S3. Scatter plots of predicted pK_d values computed from GroScores (cutout-mode, 5 independent cycles) after a linear fit vs. experimental pK_d values for the HADDOCK Protein–Protein Affinity Benchmark (46 structures) for CHARMM36/TIP3P ($pK_d = -0.0209 \times \text{GroScore} + 2.9054$).

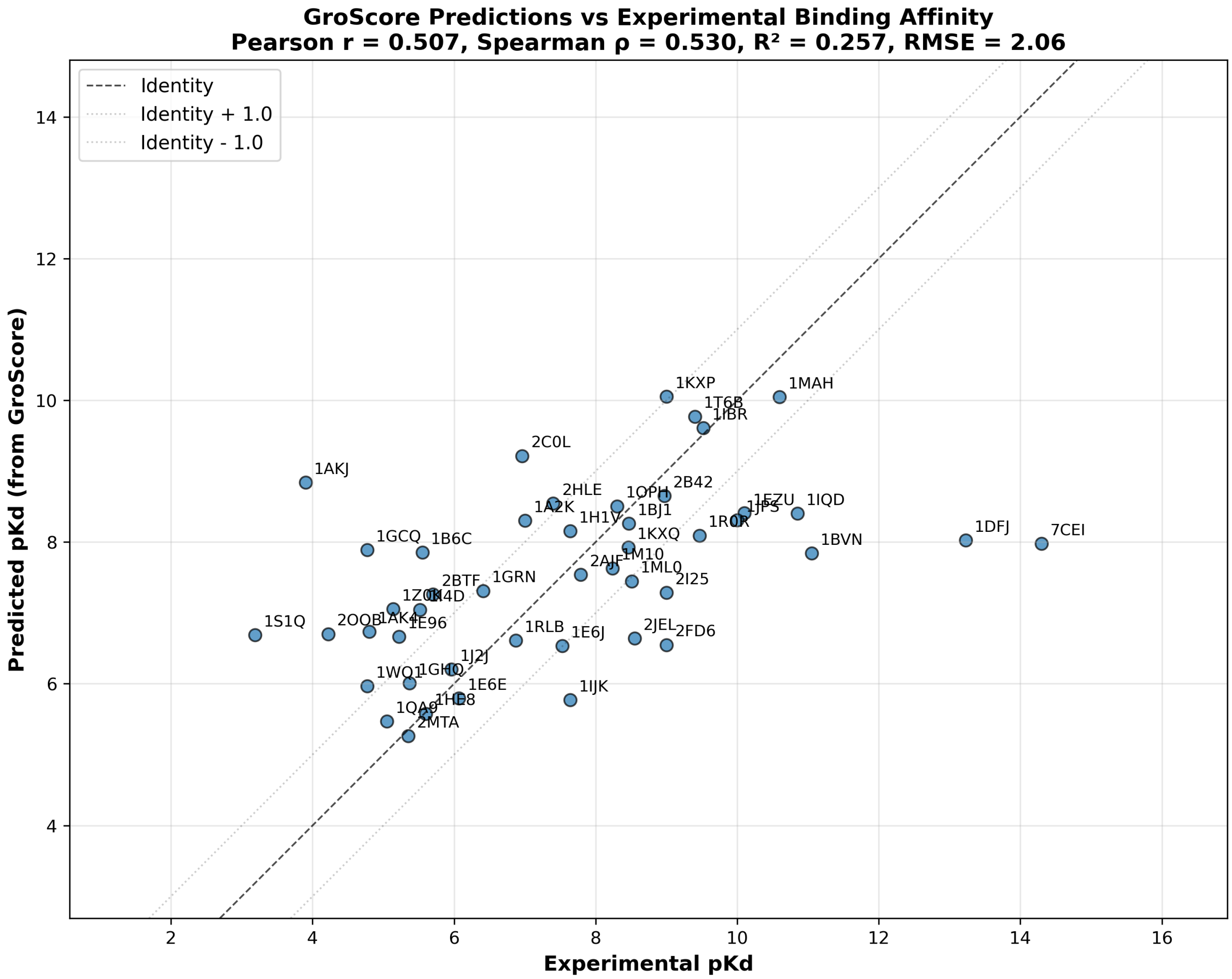

Figure S4. Scatter plots of predicted pK_d values computed from GroScores (cutout-mode, 5 independent cycles) after a linear fit vs. experimental pK_d values for the HADDOCK Protein–Protein Affinity Benchmark (46 structures) for GROMOS 54A8/SPC ($pK_d = -0.0178 \times \text{GroScore} + 3.7434$).

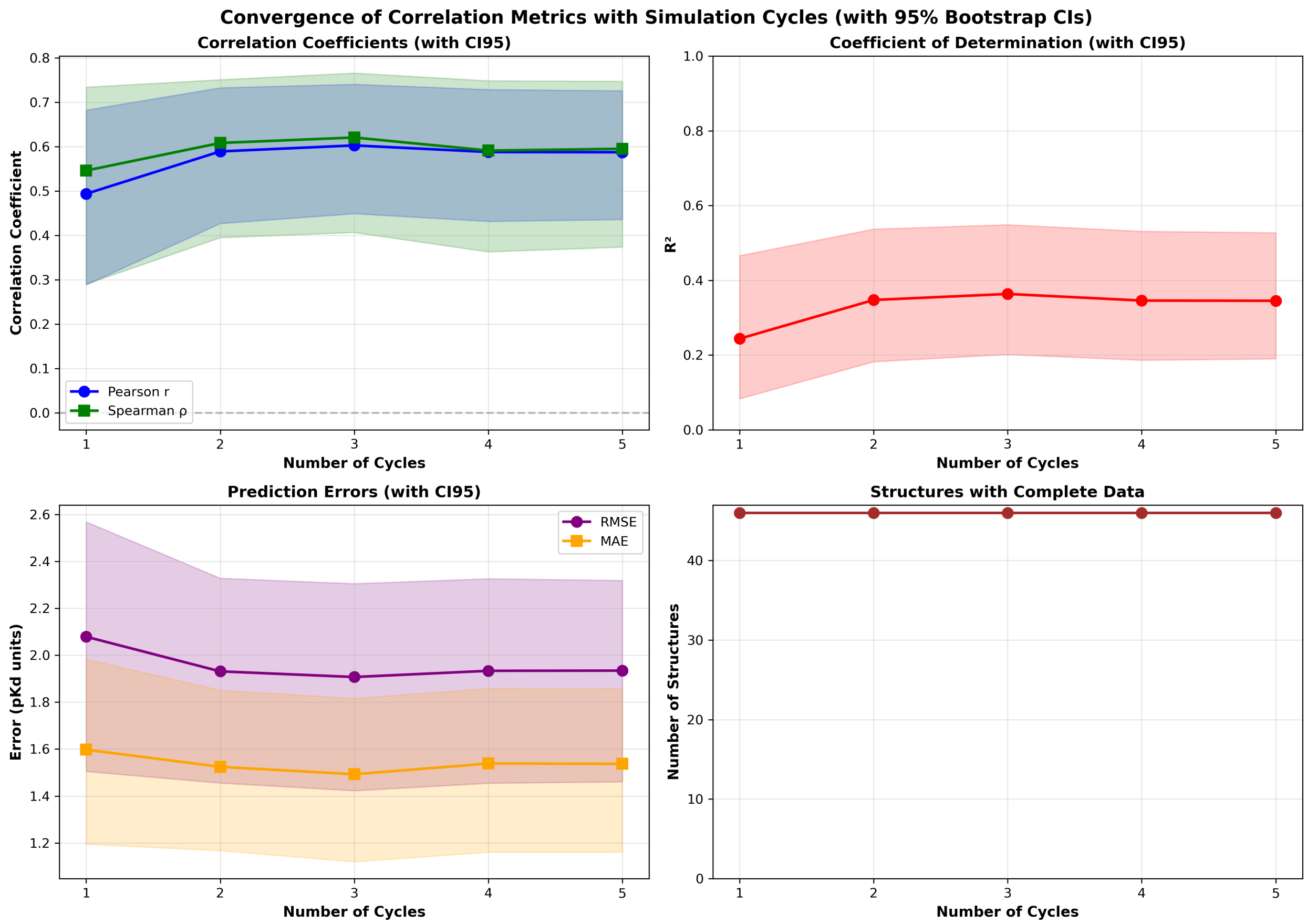

Figure S5. Convergence of GroScore (cutout-mode) prediction performance as a function of the number of independent pull/push cycles for the HADDOCK benchmark for AMBER19SB/OPC.

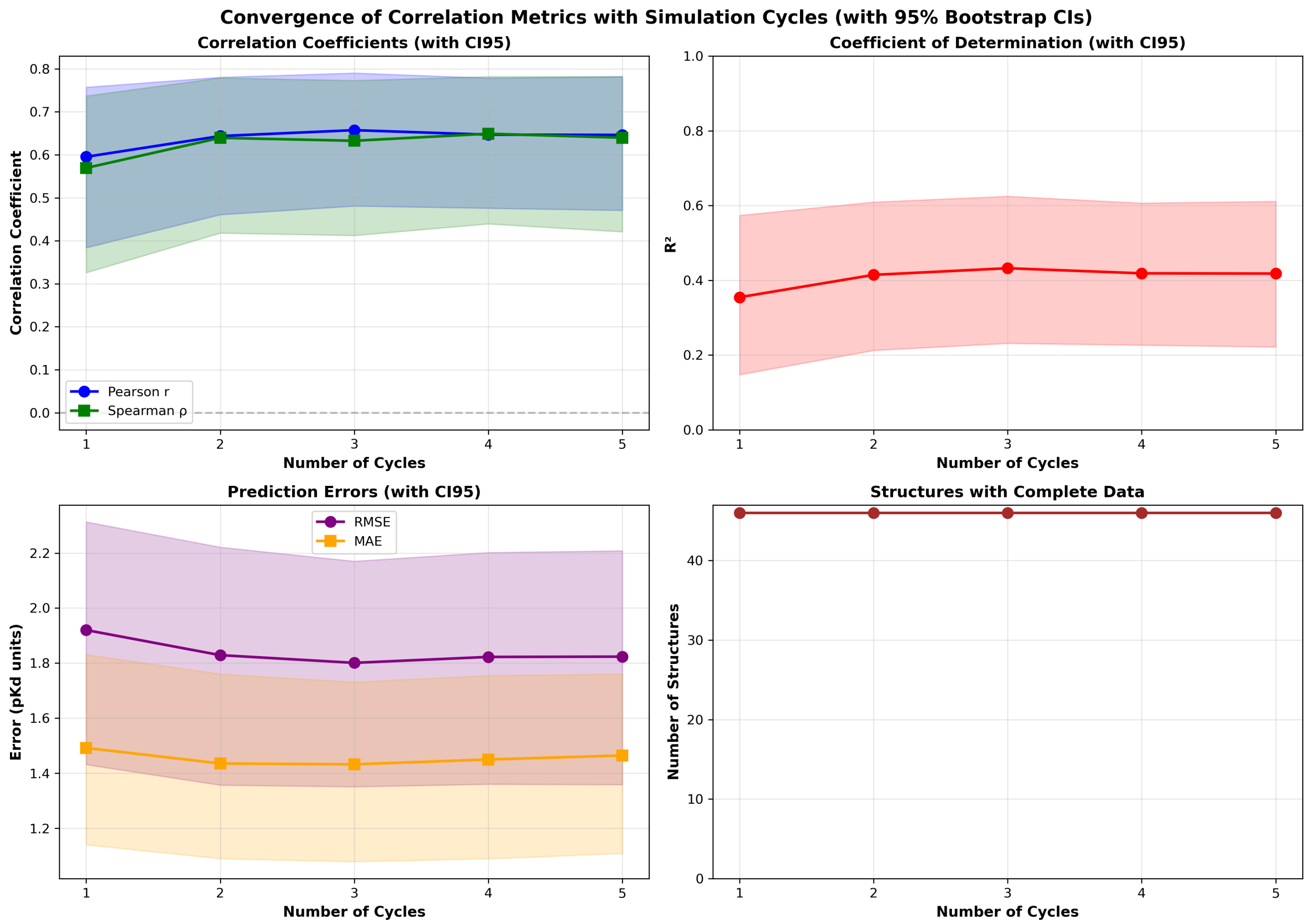

Figure S6. Convergence of GroScore (cutout-mode) prediction performance as a function of the number of independent pull/push cycles for the HADDOCK benchmark for AMBER19SB/OPC3.

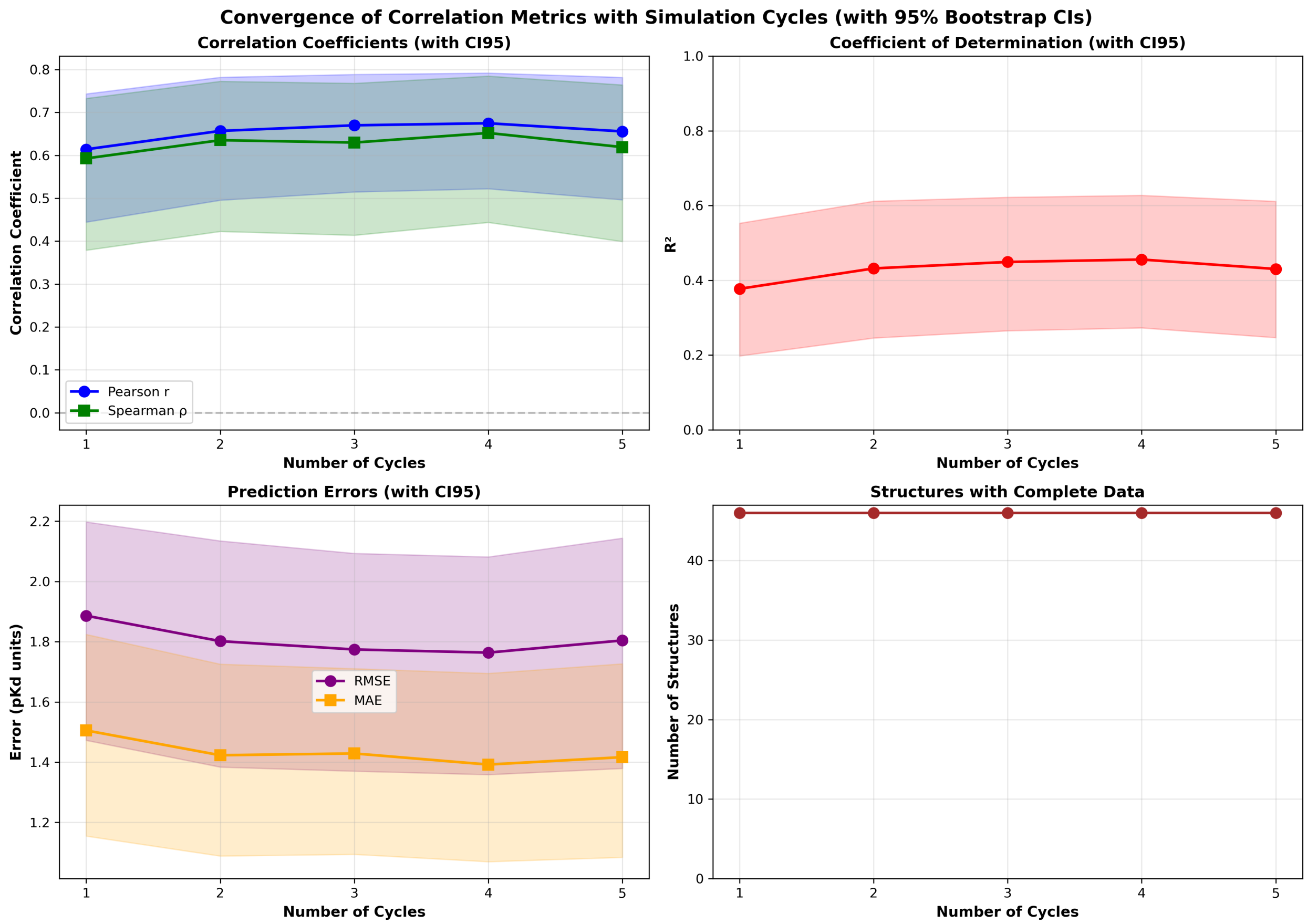

Figure S7. Convergence of GroScore (cutout-mode) prediction performance as a function of the number of independent pull/push cycles for the HADDOCK benchmark for CHARMM36/TIP3P.

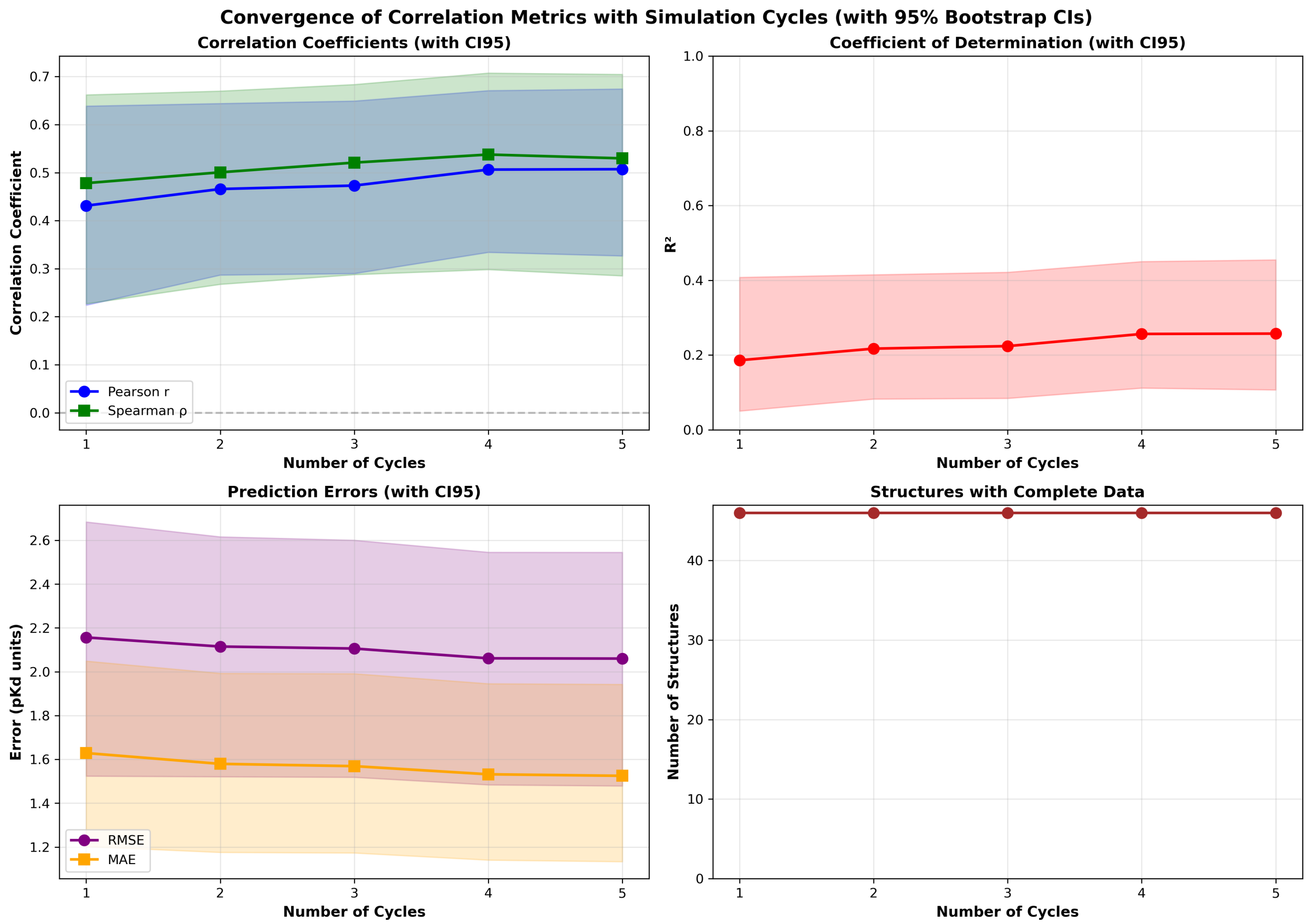

Figure S8. Convergence of GroScore (cutout-mode) prediction performance as a function of the number of independent pull/push cycles for the HADDOCK benchmark for GROMOS 54A8/SPC.

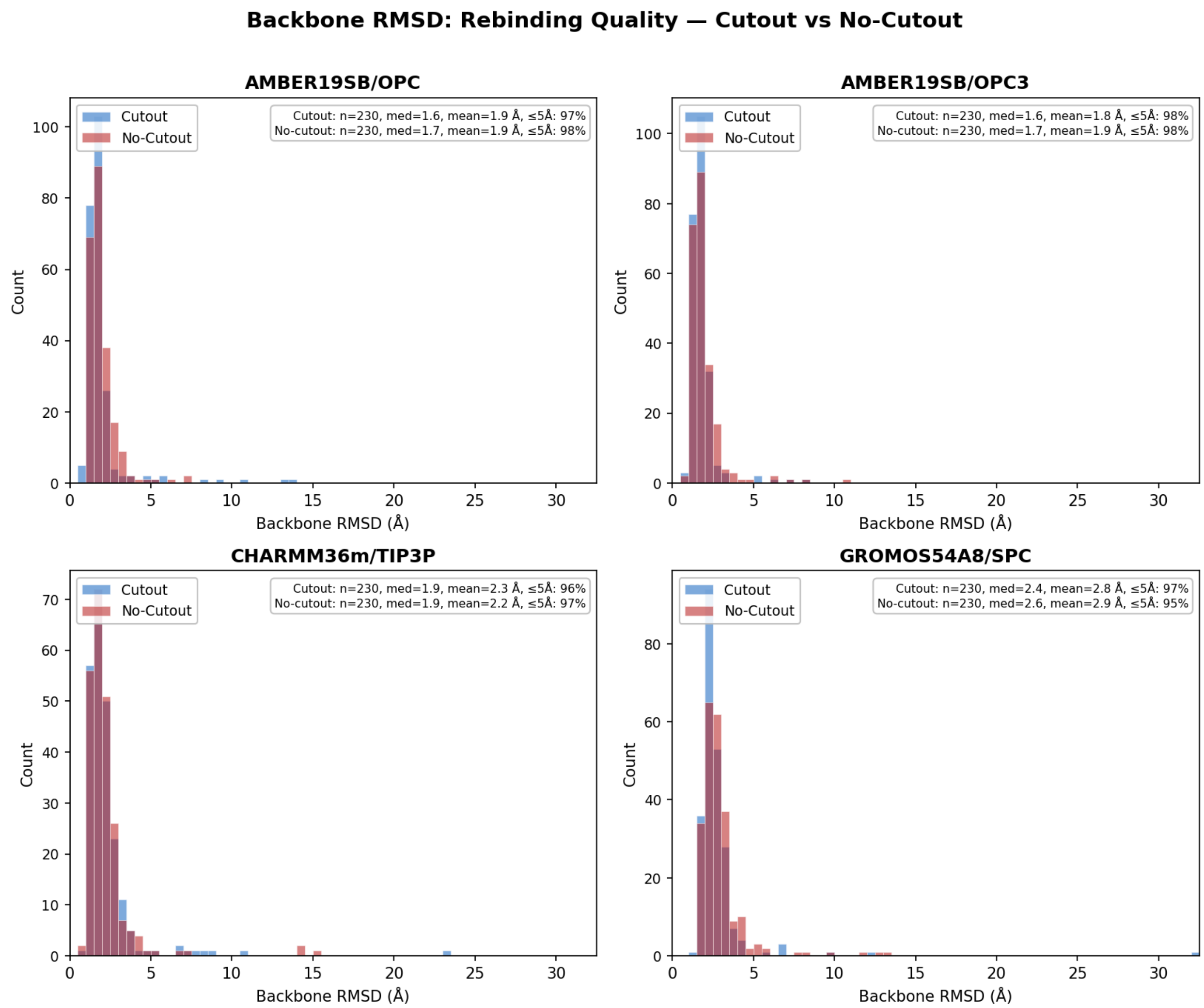

Figure S9. Backbone RMSD distributions of the rebound state relative to the initial bound state for the HADDOCK protein–protein affinity benchmark (46 complexes × 5 cycles), comparing cutout and no-cutout simulations for AMBER19SB/OPC, AMBER19SB/OPC3, CHARMM36m/TIP3P, and GROMOS 54A8/SPC. Median and mean RMSD values and the fraction of trajectories with RMSD ≤ 5 Å are given in each panel.

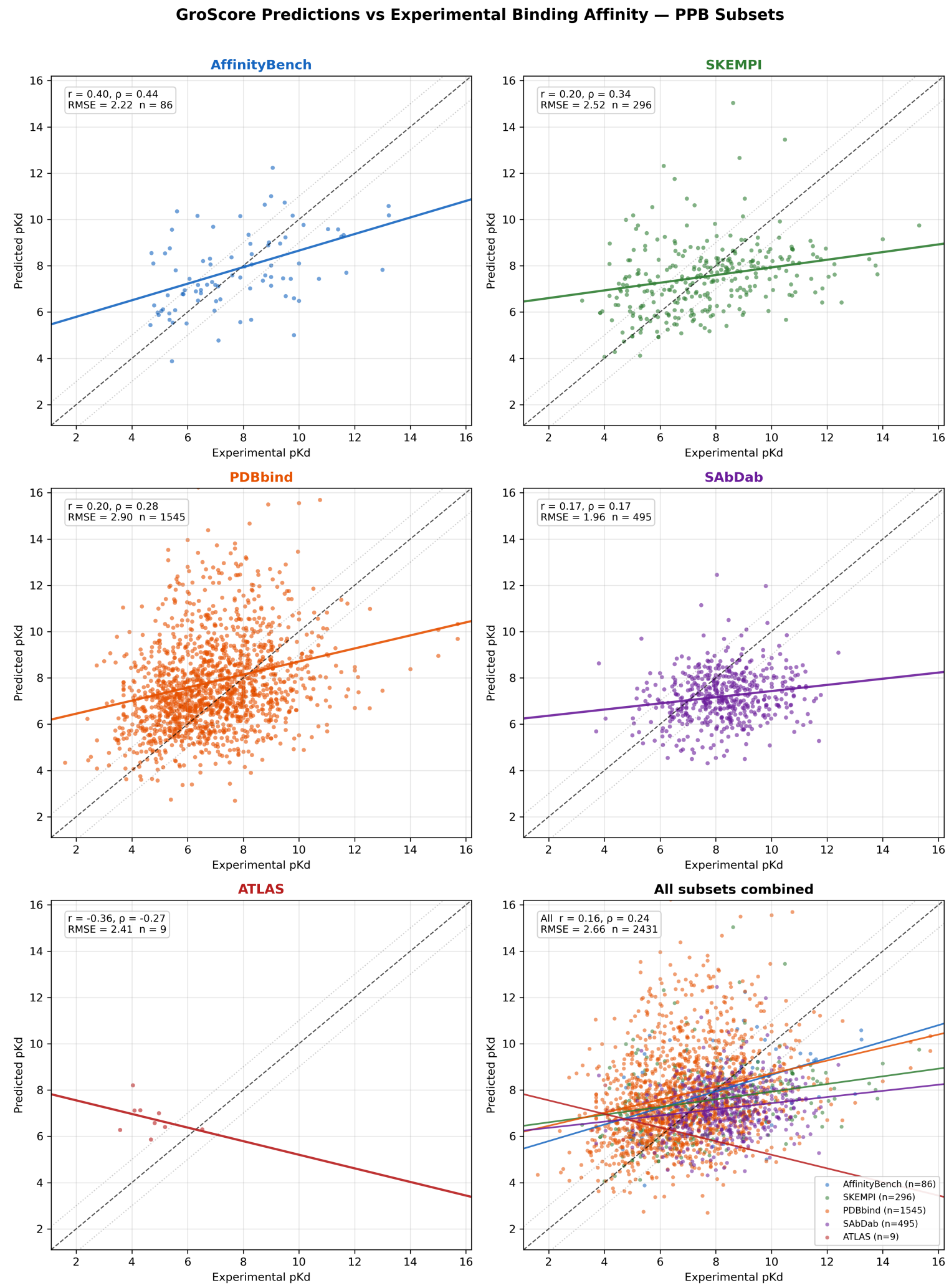

Figure S10. GroScore-predicted pK_d vs. experimental pK_d for the five PPB-Affinity source datasets. Predicted pK_d values were obtained by applying the AMBER19SB/OPC3 linear calibration determined from the HADDOCK benchmark (Figure S1) to the GroScore values. Each point represents one complex; coloured lines show subset-specific linear fits. The dashed diagonal indicates identity; dotted lines indicate identity ± 1 pK_d unit. The compressed dynamic range of predicted pK_d values relative to experiment (high-affinity complexes are systematically underpredicted and weak binders overpredicted) is consistent with the scoring method capturing relative rank order more reliably than absolute affinity. Axes were cropped for clarity; a small number of outliers fall outside the range shown.

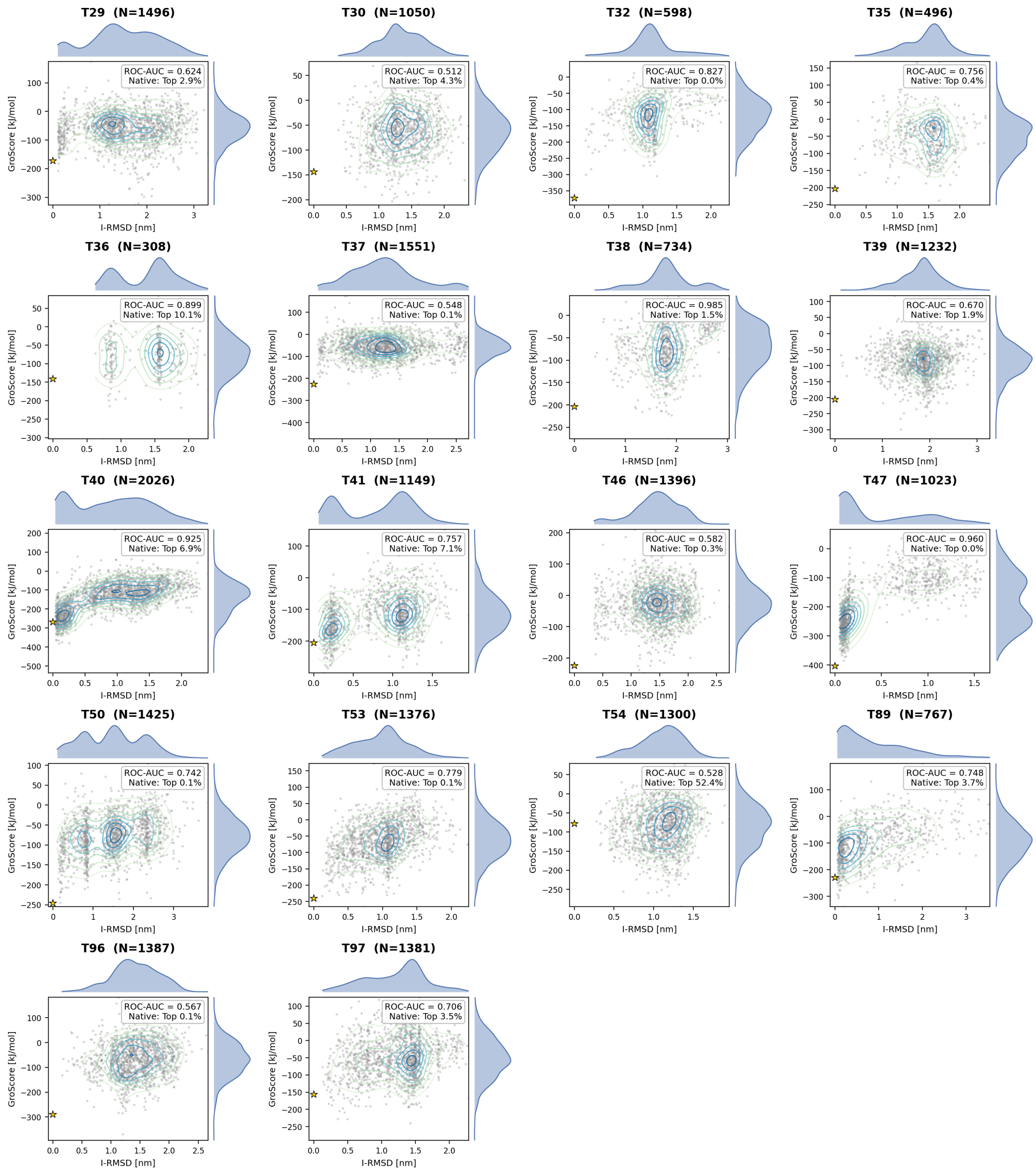

Figure S11. Scatterplots of the calculated GroScores (single-cycle scoring) against the Interface-RMSDs (I-RMSD) of the docked poses with respect to the experimentally determined structures of CAPRI Score_set targets. The scores of the native, experimentally determined structures are indicated with a star symbol. Approximate 1D and 2D distributions of Score_set values were computed using Kernel Density Estimation. ROC-AUCs were calculated considering the corresponding native structure.

Table S1. CAPRI evaluation of the Score_set benchmark (single-cycle scoring). [a] For T29 and T46, decoys with buried strands or enlaced strands were excluded from the ranking.

**Target**	**N Poses**	**N Scored**	**Natives In-Set**	**Top-10**
T29	2187	1496	167/2***/78**	1/1**[a]
T30	1344	1050	2	0
T32	599	598	15/3**	2/1**
T35	499	496	3	0
T36	309	308	0	0
T37	1700	1551	99/11***/46**	2/1**
T38	899	734	0	0
T39	1400	1232	4/3**	0
T40	2180	2026	588/193***/206**	9/5***/3**
T41	1199	1149	371/2***/120**	8/4**
T46	1700	1396	24	1[a]
T47	1051	1023	611/278***/307**	10/6***/4**
T50	1451	1425	133/36**	5/5**
T53	1400	1376	130/17**	3/1**
T54	1400	1300	19/1**	0
T89	790	767	291/35***/182**	6/2***/3**
T96	1418	1387	8/2**	0
T97	1417	1381	67/9**	2
**Total**	**22943**	**20695**	**2532/521***/1010****	**49/13***/23****
